# Stratifying metabolic-related risk factors using latent class analysis to explore the risk of renal composite endpoints in patients with type 2 diabetes mellitus and associated chronic kidney disease

**DOI:** 10.3389/fendo.2025.1599024

**Published:** 2026-01-05

**Authors:** Xiaojie Chen, Weiting He, Danfeng Liu, Runli Jia, Yaxi Zhu, Hanchen Hou, Xuan Zhao, Qijun Wan, Wenjian Wang

**Affiliations:** 1 Department of Nephrology, The First Affiliated Hospital of Shenzhen University, Shenzhen Second People’s Hospital, Shenzhen, Guangdong, China; 2 Department of Nephrology, Guangdong Provincial People’s Hospital, Guangdong Academy of Medical Sciences, Southern Medical University, Guangzhou, Guangdong, China

**Keywords:** associated chronic kidney disease, latent class analysis, metabolic-related risk factors, renal composite endpoints, type 2 diabetes mellitus

## Abstract

**Background:**

Metabolic syndrome is a key independent risk factor for the progression of chronic kidney disease (CKD) in patients with Type 2 diabetes (T2DM). Traditional studies often focus on isolated metabolic markers, but our research aims to comprehensively assess the metabolic landscape of these patients. Existing approaches have been limited in integrating multiple metabolic parameters and stratifying patients based on the severity of metabolic dysregulation, hindering the understanding of disease progression.

**Methods:**

This single-center, retrospective cohort study was conducted at Guangdong Provincial People’s Hospital, enrolling 860 participants from January 2010 to December 2023. A total of 65.0% were male, and 35.0% were female. Using Latent Class Analysis (LCA), we stratified CKD patients with T2DM into two distinct classes based on a comprehensive set of baseline clinical metabolic indicators, including glycated hemoglobin (HbA1c), lipid profiles, serum uric acid, blood pressure and body mass index (BMI). Cox proportional hazards models were used to assess renal outcome risks across these identified metabolic phenotypes.

**Results:**

LCA revealed that Class 2 exhibited significantly higher values for clinical parameters including systolic and diastolic blood pressure, BMI, total cholesterol, triglycerides, LDL-C, HbA1c, and protein-to-creatinine ratio. Longitudinal analysis showed increased hazard ratios for renal outcomes at 3, 5, and 10 years for Class 2 (HR: 1.718, 1.662, and 1.826, respectively; all P < 0.05).

**Conclusions:**

This study highlights the utility of comprehensive metabolic profiling through LCA for stratifying CKD patients with T2DM, identifying two distinct subgroups with differential renal prognoses, and offering insights for precision nephrology interventions and personalized risk management.

## Background

In the spectrum of microvascular complications associated with type 2 diabetes, concurrent chronic kidney disease (CKD) has emerged as a significant issue in contemporary nephrology. Studies show that CKD affects 20% to 40% of individuals with diabetes, making it the leading cause of end-stage renal disease (ESRD) globally (1–3). The impact of kidney disease extends beyond the decline in renal function, establishing a bidirectional relationship between kidney damage progression and increased cardiovascular risk. The progression of diabetic kidney disease severely affects patients’ quality of life. As renal function gradually declines, patients face an increased risk of end-stage renal disease (ESRD), and the associated social medical costs continue to rise. The pathogenesis of diabetic nephropathy is complex, and metabolic factors play a key role in its progression. Although the granular mechanisms linking metabolic dysregulation to chronic kidney disease remain incompletely elucidated, robust biological frameworks support this association (4). Therefore, the timely identification and therapeutic targeting of metabolism-related risk factors represents a cornerstone strategy. Early preventive measures can effectively lower chronic kidney disease (CKD) with T2DM incidence and optimize patient outcomes throughout the disease process.

Previous investigative studies have illuminated the intricate relationship between Metabolic Syndrome, the trajectory of chronic kidney disease (CKD) progression, and the manifestation of albuminuria and total proteinuria development (2, 4, 5). A comprehensive meta-analysis encompassing 24 studies and involving 6,573,911 participants revealed sophisticated revelations into this relationship. Specifically, 5 cohort studies assessed the risk of albuminuria or proteinuria in MetS patients, demonstrating a significant association (OR 1.43; 95% CI: 1.10-1.86). Stratified subgroup analyses further elucidated these findings, revealing albuminuria risk at OR 1.15 (95% CI: 1.11-1.18) and proteinuria risk at OR 1.76 (95% CI: 1.57-1.97). Additionally, 6 cohort studies investigated the risk of rapid kidney function decline among MetS patients, revealing a statistically significant correlation (OR 1.25; 95% CI: 1.07-1.47) (6).

Remarkably, disparate investigations exhibited substantial heterogeneity in risk assessment. This inherent variability in research outcomes potentially stems from multiple critical determinants: population demographic divergences, variance in follow-up durations, and region-specific and ethnic-specific contextual factors. Furthermore, the multifaceted definitional landscape of Metabolic Syndrome (MetS) may substantially influence research interpretations. Furthermore, the multifaceted definitional landscape of Metabolic Syndrome (MetS) can substantially influence research interpretations. The World Health Organization (WHO) first conceptualized insulin resistance syndrome in 1998 (7). Subsequent frameworks, such as the National Cholesterol Education Program Adult Treatment Panel III (NCEP-ATP III) (8). and the International Diabetes Federation (IDF), introduced evolving definitions, with the IDF in 2005 (9) emphasizing abdominal adiposity as a key component. By 2009, a collaborative consortium established a unified MetS standard (10). The MetS diagnostic framework encompasses critical metabolic parameters: blood pressure, glucose, metabolism, lipid profiles, and obesity, all these factors are linked to chronic kidney disease progression. Notably, while serum uric acid remains excluded from primary MetS diagnostic criteria, it is increasingly recognized as a significant metabolic biomarker. Although serum uric acid is not included in the classic definition of metabolic syndrome, numerous studies have shown that it plays an important role in metabolic syndrome. For example, in the genetic study by Yang (11), metabolic factors such as BMI, high-density lipoprotein (HDL), and systolic blood pressure (SBP) were found to have a significant causal relationship with serum urate levels and gout risk. Furthermore, there is a bidirectional causal effect between serum urate and metabolic traits, with hyperuricemia being closely associated with obesity, insulin resistance, and other metabolic issues. Therefore, in this study, we have included serum urate as a key metabolic indicator in the metabolic grouping of patients with diabetic nephropathy to more comprehensively assess its relationship with metabolic risk and kidney disease progression.

The distinctive contribution of our investigation lies in innovatively employing Latent Class Analysis (LCA). Unlike traditional regression techniques, LCA represents a sophisticated mixed modeling approach designed to identify the most optimal data representation through the premise of unobserved heterogeneous subgroups within the dataset. Currently no studies have used latent class analysis to identify a subgroup of T2DM associated CKD patients at high risk for progression of renal deterioration due to metabolic disorders. Therefore, by integrating metabolic markers blood pressure, glycemic profiles, lipid metabolism, body mass index, and serum uric acid, our study aimed to identify a potentially high-risk subgroup of metabolic disorders in patients with T2DM associated CKD patients and to assess their risk of renal function progression during short-term, intermediate, and long-term follow-up periods.

## Materials and methods

### Study population and design

This study was a single-center retrospective cohort study. The patients were recruited from the outpatient and inpatient departments of Guangdong Provincial People’s Hospital from January 2010 to December 2023.

The enrollment criteria of patients were as follows: 1) Clinically diagnosed as type 2 diabetic nephropathy (12); 2) Patients aged 18–80 years; 3) At least 2 follow-up visits.

Exclusion criteria were as follows: 1) Patients were followed up for less than 3 months; 2) Patients with baseline eGFR less than 15 ml/min/1.73 m²; 3) Patients had undergone hemodialysis, peritoneal dialysis, and kidney transplantation at the first visit; 4) Patients with malignant tumors, infection, pregnancy, etc.

In this study, we used Latent Class Analysis (LCA) to identify latent subgroups of patients with chronic kidney disease (CKD) combined with type 2 diabetes. To evaluate different numbers of latent classes, we constructed multiple LCA models and assessed them using the Akaike Information Criterion (AIC) and Bayesian Information Criterion (BIC). AIC and BIC were used to balance model fit and complexity, avoiding both overfitting and underfitting. We started with a two-class model and progressively increased the number of classes (up to five), comparing the AIC and BIC values of each model (**Table 1**). Ultimately, we chose the two-class model because it provided the best balance between AIC and BIC values while offering clinically meaningful subgroupings. Specifically, the two-class model showed good posterior probabilities and had moderate subgroup sizes (**Table 2**), ensuring both stability and clinical applicability of the results.

**Table 1:** Evaluation of latent class models: AIC and BIC metrics.

Number of class	AIC	BIC	N1	N2	N3	N4	N5
2	20516.4	20697.2	284(33%)	576(67%)			
3	20203.4	19927.5	353(41%)	293(34.1%)	214(24.9%)		
4	19591.2	19962.1	114(13.3%)	312(36.3%)	208(24.2%)	226(26.3%)	
5	19273.6	19739.8	215 (25%)	166 (19.3%)	84 (9.8%)	233 (27.1%)	162 (18.8%)

AIC, Akaike information criterion; BIC, Bayesian Information Criterion; N, number.

**Table 2:** Posterior probabilities in two-class model.

Class	1	2
1	0.9286 (0.5005 - 1.0000)	0.0493 (0.0001 - 0.4998)
2	0.0714 (<0.0001 - 0.4995)	0.9507 (0.5002 - 0.9999)

The Medical Research Ethics Committee of Guangdong Provincial People’s Hospital (Guangdong Academy of Medical Sciences) approved the retrospective study project (NO.GDREC2017205H (R1)).

**Figure 1:**
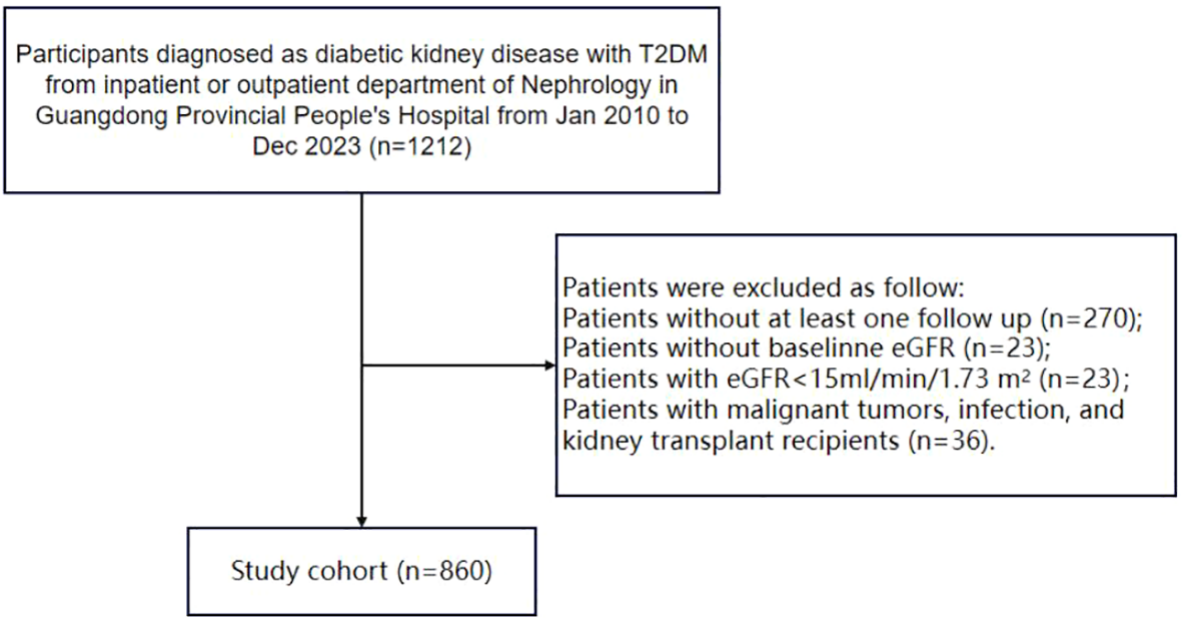
Flowchart of the study.

**Figure 1** illustrates the patients selection process in the study, with a total of 860 patients ultimately included in this research.

### Outcomes

The outcomes included two renal endpoints: a decline of more than 50% from baseline estimated Glomerular Filtration Rate (eGFR) or an eGFR of less than 15 ml/min/1.73 m². The remaining patients were followed up until December 31st, 2023. The outcomes were categorized as a dichotomous variable, where 0 indicated that the patient did not reach the renal endpoint events, and 1 indicated that the patient did reach the renal endpoint events.

### Ascertainment of type 2 diabetes mellitus associated chronic kidney disease

Type 2 Diabetes Mellitus associated Chronic Kidney Disease refers to a chronic kidney disease that occurs in patients with type 2 diabetes, characterized by a persistent increase in urinary albumin excretion (urinary albumin-to-creatinine ratio >30 mg/g) and/or a reduction in the estimated glomerular filtration rate (eGFR) to <60 ml/min/1.73 m² (13, 14).

### Assessment of covariates

We selected the covariates based on our clinical experience and existing literature. The covariates included: (1) continuous variables: age, body mass index (BMI), systolic blood pressure (SBP), diastolic blood pressure (DBP), total cholesterol (TC), high-density lipoprotein cholesterol (HDL-C), low-density lipoprotein cholesterol (LDL-C), triglycerides (TG), serum creatinine (Scr), uric acid (UA), albumin (ALB), glycosylated hemoglobin (HbA1c), Urine Protein-to-Creatinine Ratio (PCR), and Urine Albumin-to-Creatinine Ratio (ACR); (2) categorical variables: gender.

Demographic information, including age and gender, was collected during each hospital visit. Trained staff measured height, weight, and blood pressure to ensure accuracy. Body Mass Index (BMI) was calculated using the formula: weight in kilograms divided by height in meters squared (kg/m²). The estimated Glomerular Filtration Rate (eGFR) value was calculated from age (in years), sex, and serum creatinine (in mg/dL), using the Chronic Kidney Disease Epidemiology Collaboration equation (15). Blood pressure measurements were taken using standard mercury sphygmomanometers, which are recognized for their reliability. The baseline of follow-up was established by the time when clinicians initially diagnosed the participants with type 2 diabetic kidney disease. Subsequently, throughout the patient follow-up period, renal function changes were systematically assessed and monitored.

### Ethics approval

All individuals provided written informed consent after being fully briefed and advised about the study procedures. This study was approved by the Ethics Committee of Guangdong Provincial People’s Hospital. It was conducted in compliance with the Declaration of Helsinki. The Medical Research Ethics Committee of Guangdong Provincial People’s Hospital (Guangdong Academy of Medical Sciences) approved this retrospective study (NO.GDREC2017205H(R1)).

### Statistical analysis

This study employed Latent Class Analysis (LCA) to identify clinical subtypes within a cohort of patients with Type 2 Diabetic Kidney Disease (DKD). LCA is a statistical method based on finite mixture model theory, which assumes that the observed multivariate distribution is composed of mixtures from multiple latent populations (16). Compared to traditional univariate classification methods, LCA’s advantage lies in its ability to simultaneously integrate multiple clinical indicators for unsupervised classification, thereby obtaining disease subtypes with greater clinical significance (17). The patients participating in this study were diagnosed with Type 2 Diabetic Kidney Disease by experienced clinicians from the Department of Nephrology at Guangdong Provincial People’s Hospital, providing rich clinical and demographic variable data with minimal missing data. The patient data was from both inpatient and outpatient medical records. We explored models identifying 2 to 4 categories, comparing them using the Bayesian Information Criterion(BIC) and Akaike Information Criterion(AIC) (18).

Our study identified metabolically high-risk groups for composite renal endpoints during 3-year, 5-year, and 10-year follow-up periods. Latent Class Analysis (LCA) evaluated individual participants’ baseline characteristics and generated participant categories based on their baseline characteristic patterns. Using LCA, we assigned all patients to non-overlapping categories in a data-driven manner, meaning each patient was assigned to only one category (19–21). The indicators included in our study were age, gender, Body Mass Index (BMI), Systolic Blood Pressure (SBP), Diastolic Blood Pressure (DBP), glycated hemoglobin (HbA1c), Total Cholesterol (TC), Triglycerides (TG), Low-Density Lipoprotein Cholesterol (LDL-C), High-Density Lipoprotein Cholesterol (HDL-C), Serum Creatinine (Scr), estimated Glomerular Filtration Rate (eGFR), serum uric acid (UA), hemoglobin (Hb), and albumin (Alb). In our study, patients’ baseline metabolic-related factors were considered as category-defining indicators in the LCA model. Based on current guidelines’ varying definitions of metabolic syndrome (7, 22, 23), we selected systolic blood pressure, diastolic blood pressure, glycated hemoglobin, blood lipids (total cholesterol, triglycerides, low-density lipoprotein cholesterol, high-density lipoprotein cholesterol), and BMI as our grouping criteria. Additionally, as serum uric acid is increasingly recognized as a metabolism-related risk factor, we also included it as a classification indicator. We employed a progressive approach for Latent Class Analysis (LCA), starting with k=1 category and gradually increasing the number of categories. Drawing from previous research experience and to ensure clinical practicality, we set the minimum sample size for each category at no less than 1.5% of the total study population (24). During model construction, posterior probabilities were used as key indicators for evaluating category assignment and individual classification (25). Posterior probability reflects the probability estimate of an individual belonging to a specific latent category given the observed data. Specifically, the LCA model first establishes a mathematical model based on observed variables (including demographic characteristics, clinical indicators, etc.), then calculates the conditional probability of each participant belonging to each latent category. These probability estimates are derived from comprehensive calculations based on model parameters and individual observational data. In the actual classification process, participants were assigned to the category with the highest posterior probability. The distribution characteristics of posterior probabilities can also be used to evaluate the model’s goodness of fit; that is, if most participants’ posterior probabilities show high concentration in a particular category, it indicates that the classification scheme has good discriminative ability (26). Through systematic analysis of posterior probability distributions, we can gain a deeper understanding of the characteristics of various latent classes, providing scientific evidence for subsequent clinical decision-making and intervention strategy development. Many studies set a posterior probability of 0.7 or higher as the criterion for assigning participants to specific categories (27–29). This means that participants have a high probability of belonging to that category, indicating strong confidence in their category assignment. For the best-fit category, posterior probabilities approaching 1, while approaching 0 for other categories, indicate high classification certainty. In our study, we stopped fitting additional categories when the model’s posterior probability fell below 85%. This cutoff value was chosen to ensure higher accuracy of latent classes and reliability when designing category-specific preventive strategies. In determining the optimal number of classes for our Latent Class Analysis (LCA) model, we implemented a comprehensive multi-criteria evaluation framework. The initial selection process was guided by model fitness indicators, with particular emphasis on two fundamental information criteria: the Akaike Information Criterion (AIC) and the Bayesian Information Criterion (BIC). The interpretative principle for these metrics follows an inverse relationship - the lower the numerical values of these information criteria, the stronger the model’s goodness of fit to the observed data patterns (30–32). However, the final determination of class numbers was beyond purely statistical metrics, incorporating a synergistic evaluation that balanced quantitative fitting indices with accumulated clinical expertise and practical insights. After determining the definitive number of classes, we employed a nuanced modeling approach to examine the associations between class membership and outcome variables. This analytical framework was specifically designed to accommodate the inherent uncertainty that individuals may exhibit in their class assignments, therefore providing a more robust and realistic assessment of the relationships under investigation.

To describe baseline characteristics of the total participants and different categories, two-sample t-tests were used for normally distributed continuous variables, Wilcoxon rank-sum tests for non-normally distributed continuous variables, and chi-square tests for categorical variables. Continuous variables were presented as mean ± standard deviation (for normal distribution) or median (interquartile range) (for skewed distribution), while categorical variables were expressed as frequencies or percentages. Additionally, chi-square tests were employed to compare the incidence of composite renal endpoints among different categories. The Kaplan-Meier method was used to compare survival estimates and cumulative event rates based on time to first event. Univariate and multivariate Cox proportional hazards regression analyses were applied to compare the risks of composite renal endpoints among different categories at 3, 5, and 10 years, with hazard ratios (HR) and 95% confidence intervals (CI) used to assess the risk of composite renal endpoints. Simultaneously, log-rank tests were used to compare the hazard ratios (HR) of adverse events in Kaplan-Meier analyses. All results were reported in accordance with the STROBE statement (33).

All analyses were performed using the statistical software package R (http://www.R-project.org, R Foundation), IBM SPSS Statistics software (version 27.0; IBM Corporation, Armonk, NY), and Empower-Stats (http://www.empowerstats.com, X&Y Solutions, Inc., Boston, Massachusetts). All tests were two-tailed, and P < 0.05 was considered statistically significant.

## Results

### Latent-class modeling

We conducted an iterative latent class analysis, examining models with one through five classes (k1-k5). The model fit statistics for solutions containing two to five latent classes are summarized in **Table 1**. All identified classes maintained a minimum size threshold exceeding 1.5% of the study population, ensuring practical significance. Both Akaike Information Criterion (AIC) and Bayesian Information Criterion (BIC) demonstrated progressive improvement with increasing class numbers, suggesting that the additional model complexity was justified by enhanced fit.

The posterior probabilities for each model solution were calculated to assess classification precision. All identified classes across the four models (k2-k5) exhibited posterior probabilities exceeding the threshold of 85%. In the two-class solution (**Table 2**), both classes demonstrated robust classification accuracy, with posterior probabilities of 0.9286 (0.5005-1.0000) and 0.9507 (0.5002-0.9999) for Classes 1 and 2, respectively. The three-class solution (**Supplementary Table 1**) maintained strong classification performance, with posterior probabilities of 0.9053 (0.5108-0.9999), 0.9273 (0.4944-1.0000), and 0.9470 (0.5167-1.0000) for Classes 1, 2, and 3, respectively.

However, classification precision showed notable deterioration in more complex solutions. The four-class model (**Supplementary Table 1**) revealed suboptimal performance for Classes 2 and 3, with posterior probabilities falling below 90% [0.8931 (0.3919-0.9988) and 0.8975 (0.5027-0.9999), respectively]. Similarly, the five-class solution (**Supplementary Table 1**) exhibited reduced classification accuracy, particularly for Classes 2 and 4 [0.8600 (0.4141-1.0000) and 0.8500 (0.3958-0.9991), respectively]. Based on these classification metrics, the two-class and three-class solutions demonstrated superior classification accuracy compared to the more complex four-class and five-class models.

### Baseline characteristics of participants

A total of 860 eligible participants were enrolled in this study, with a gender distribution of 65.0% male and 35.0% female. In the two-class model, 586 participants (68.14%) were classified into Class 1 and 274 (31.86%) into Class 2. The demographic and clinical characteristics of the study population were as follows: The mean age was 56.48 ± 9.81 years, and the average body mass index (BMI) was 25.28 ± 3.77 kg/m². Blood pressure measurements revealed mean systolic (SBP) and diastolic blood pressure (DBP) values of 147.68 ± 24.67 mmHg and 84.29 ± 13.12 mmHg, respectively. Regarding biochemical parameters, the mean hemoglobin (Hb) concentration was 120.81 ± 22.90 g/L, and serum albumin averaged 35.79 ± 7.13 g/L. The lipid profile showed mean values of total cholesterol (TC) at 5.58 ± 1.98 mmol/L, low-density lipoprotein cholesterol (LDL-C) at 3.40 ± 1.34 mmol/L, triglycerides (TG) at 2.38 ± 1.86 mmol/L, and high-density lipoprotein cholesterol (HDL-C) at 1.14 ± 0.35 mmol/L. The mean glycated hemoglobin (HbA1c) was 8.42 ± 2.21%. Renal function parameters indicated a mean serum creatinine (Scr) of 138.35 ± 72.34 μmol/L and an estimated glomerular filtration rate (eGFR) of 58.25 ± 30.13 ml/min/1.73m². The baseline urinary protein-to-creatinine ratio (PCR) and albumin-to-creatinine ratio (ACR) were 2591.98 ± 4087.24 mg/g and 1516.06 ± 2007.63 mg/g, respectively. The mean serum uric acid (UA) level was 423.12 ± 118.22 mmol/L.

We sought to understand the demographic and clinical characteristics distinguishing between groups in the two-class model. Our analysis revealed that Class 2 participants demonstrated significantly higher values in several clinical parameters, including systolic blood pressure (SBP), diastolic blood pressure (DBP), body mass index (BMI), total cholesterol (TC), triglycerides (TG), low-density lipoprotein cholesterol (LDL-C), hemoglobin (Hb), glycated hemoglobin (HbA1c), protein-to-creatinine ratio (PCR), and albumin-to-creatinine ratio (ACR). However, Class 2 patients exhibited lower serum albumin (Alb) levels. Demographically, Class 2 was characterized by a lower proportion of males and younger age. (**Table 3**).

**Table 3:** Characteristics at baseline for each class in the dual-class framework.

Characteristics	Class 1	Class 2	Standardize diff.	P-value
N	586	274		
SBP(mmHg)	143.692 ± 22.952	156.212 ± 26.056	0.510 (0.364, 0.655)	<0.001
DBP(mmHg)	82.424 ± 12.059	88.277 ± 14.378	0.441 (0.296, 0.586)	<0.001
BMI(kg/m^2^)	24.674 ± 3.496	26.563 ± 4.005	0.503 (0.357, 0.648)	<0.001
Age(yr)	57.063 ± 9.370	55.226 ± 10.614	0.183 (0.040, 0.327)	0.010
Hb(g/L)	119.514 ± 23.522	123.586 ± 21.295	0.181 (0.038, 0.325)	0.015
UA(umol/L)	420.478 ± 122.774	428.773 ± 107.830	0.072 (-0.072, 0.215)	0.338
TC(mmol/L)	4.765 ± 1.230	7.157 ± 2.272	1.309 (1.153, 1.465)	<0.001
TG(mmol/L)	1.582 ± 0.666	4.082 ± 2.376	1.433 (1.274, 1.592)	<0.001
LDL-C(mmol/L)	2.939 ± 0.880	4.384 ± 1.595	1.122 (0.969, 1.275)	<0.001
HDL-C(mmol/L)	1.121 ± 0.325	1.172 ± 0.395	0.141 (-0.003, 0.284)	0.050
Alb(g/L)	36.581 ± 6.863	34.090 ± 7.414	0.349 (0.204, 0.493)	<0.001
HbA1c(%)	8.038 ± 1.905	9.241 ± 2.574	0.531 (0.386, 0.677)	<0.001
Follow-up days	643.298 ± 544.962	586.469 ± 538.115	0.105 (-0.039, 0.249)	0.154
eGFR(ml/min/1.73m²)	58.341 ± 29.763	58.065 ± 30.966	0.009 (-0.134, 0.153)	0.900
Gender			0.157 (0.014, 0.301)	0.031
Male	395 (67.406%)	164 (59.854%)		
Female	191 (32.594%)	110 (40.146%)		
PCR(mgl/g)	1942.579 ± 2721.077	3980.848 ± 5818.647	0.449 (0.304, 0.594)	<0.001
ACR(mgl/g)	1192.021 ± 1637.881	2188.566 ± 2485.358	0.473 (0.321, 0.626)	<0.001
50% eGFR decline			0.215 (0.071, 0.359)	0.003
No	493 (84.130%)	207 (75.547%)		
Yes	93 (15.870%)	67 (24.453%)		
Proceed to ESRD			0.160 (0.017, 0.304)	0.025
No	498 (84.983%)	216 (78.832%)		
Yes	88 (15.017%)	58 (21.168%)		
Composite Endpoint			0.202 (0.058, 0.345)	0.005
No	460 (78.498%)	191 (69.708%)		
Yes	126 (21.502%)	83 (30.292%)		

Values are n (%) or mean ± SD. SBP, Systolic blood pressure; DBP, Diastolic blood pressure; BMI, Body mass index; Hb, Hemoglobin; UA, Uric Acid; TC, Total cholesterol; TG, Triglyceride; LDL-C, Low density lipid cholesterol; HDL-C, High density lipoprotein cholesterol; Alb, Albumin, HbA1c, Glycated Hemoglobin, eGFR, Estimated Glomerular Filtration Rate; PCR, Protein-Creatinine Ratio; ACR, Albumin-Creatinine Ratio; ESRD, End-Stage Renal Disease.

**Figure 2:**
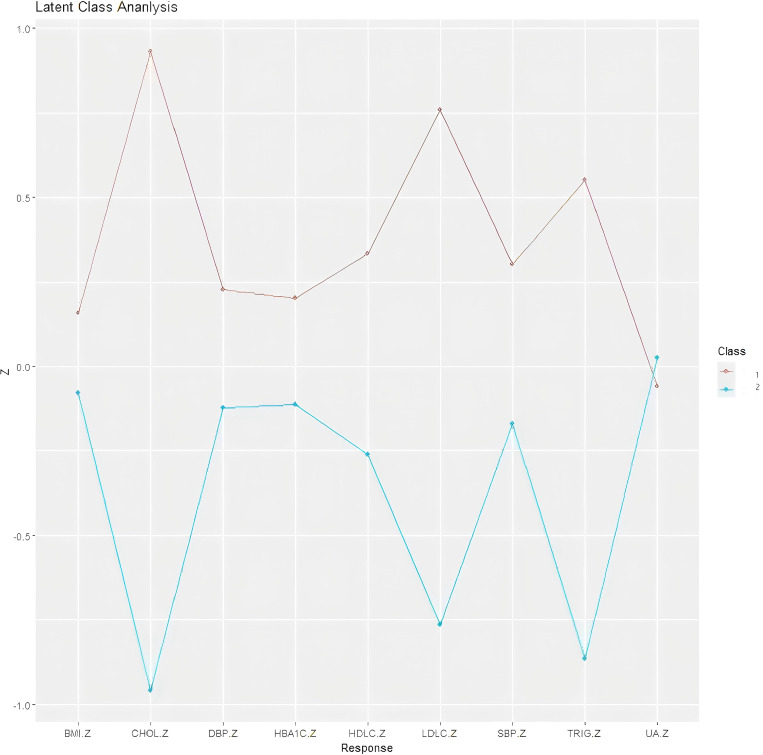
This figure demonstrates the distribution of standardized values across different variables in the two-class model, effectively illustrating the characteristic differences between classes across various parameters. The y-axis represents the standardized values, while the x-axis displays the individual continuous variables. In the variable standardization process, all variables were transformed such that the overall mean was adjusted to 0 and the standard deviation to 1. Consequently, when a standardized variable value equals +1 for a particular class, it indicates that the mean value of that variable in the given class is one standard deviation higher than the overall mean.

**Figure 2** illustrates the differences in continuous variables between the two classes. Compared to Class 1, Class 2 exhibited distinct metabolic characteristics, including elevated blood pressure (both SBP and DBP), higher BMI, increased lipid levels (TG, TC, and LDL-C), and higher HbA1c. However, no significant differences were observed between the two classes in metabolic indicators HDL-C and UA. Among all metabolic parameters, the three indicators showing the greatest disparities between classes were lipid-related measures: LDL-C, TC, and TG.

**Figure 3:**
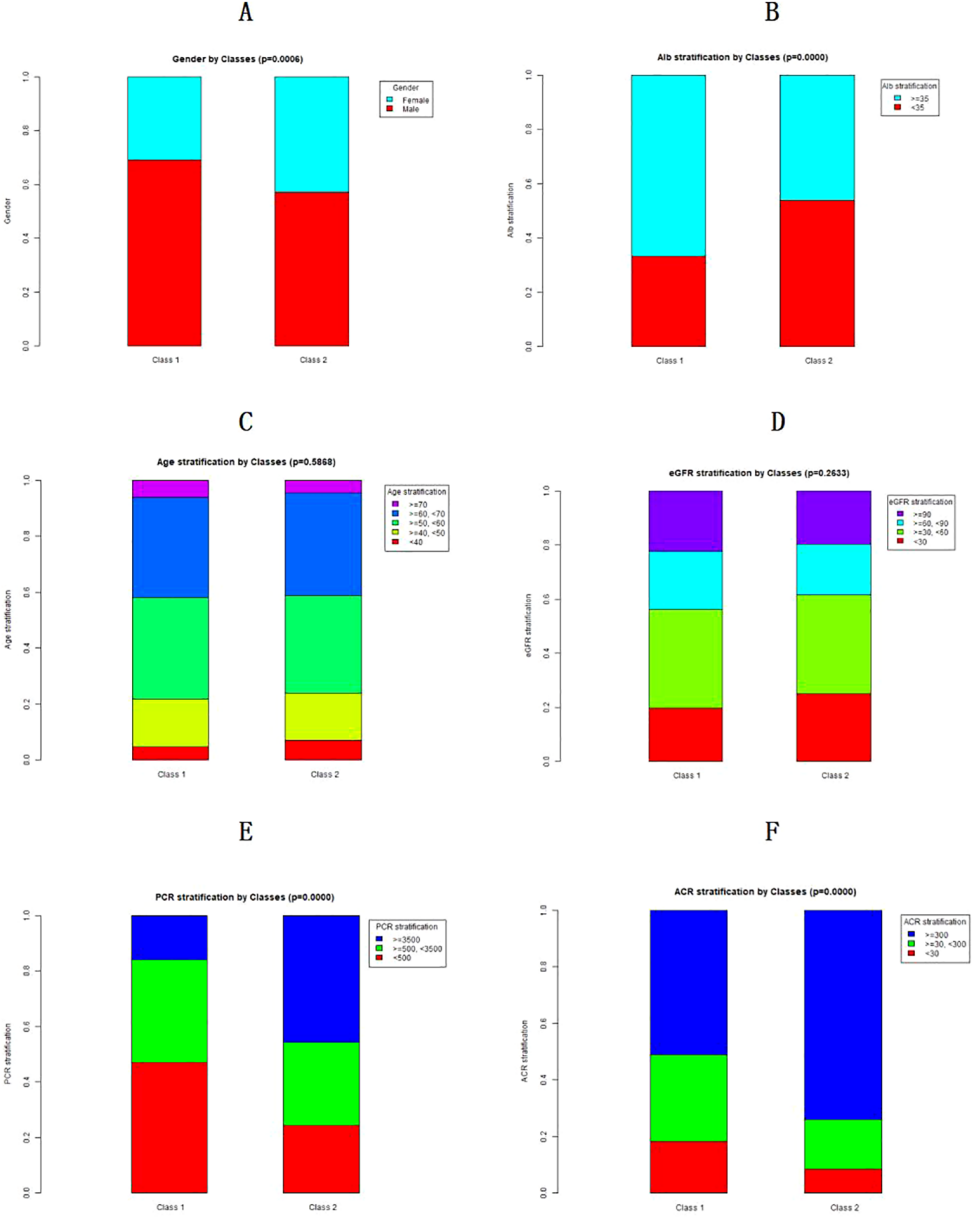
Differences in patient classification variables within the two-category model. **(A)** shows the differences in gender distribution between the two categories. **(B)** presents the differences in albumin levels across the two-category model. **(C)** highlights the differences in baseline CKD stages among the two patient categories. **(D)** depicts the differences in age groups between the two categories of patients. **(E)** depicts the differences in PCR stratification between the two categories of patients. **(F)** presents the differences in ACR stratification between the two categories of patients.

**Figure 3** demonstrates the differences in categorical variables (gender, albumin ≥35g/L or <35g/L, CKD staging, and age groups) between the two classes. Compared to Class 1, Class 2 showed a significantly higher proportion of females (P = 0.0006), a greater percentage of patients with albumin <35g/L (P<0.001), and higher quantitative values of PCR and ACR (P<0.001) (see **Figures 3A, B, E, F**). However, no statistically significant differences were observed between Class 1 and Class 2 patients in age distribution (P = 0.5868) or baseline CKD staging (P = 0.2633) (see **Figures 3C, D**).

### Association between classes and outcomes

**Figure 4:**
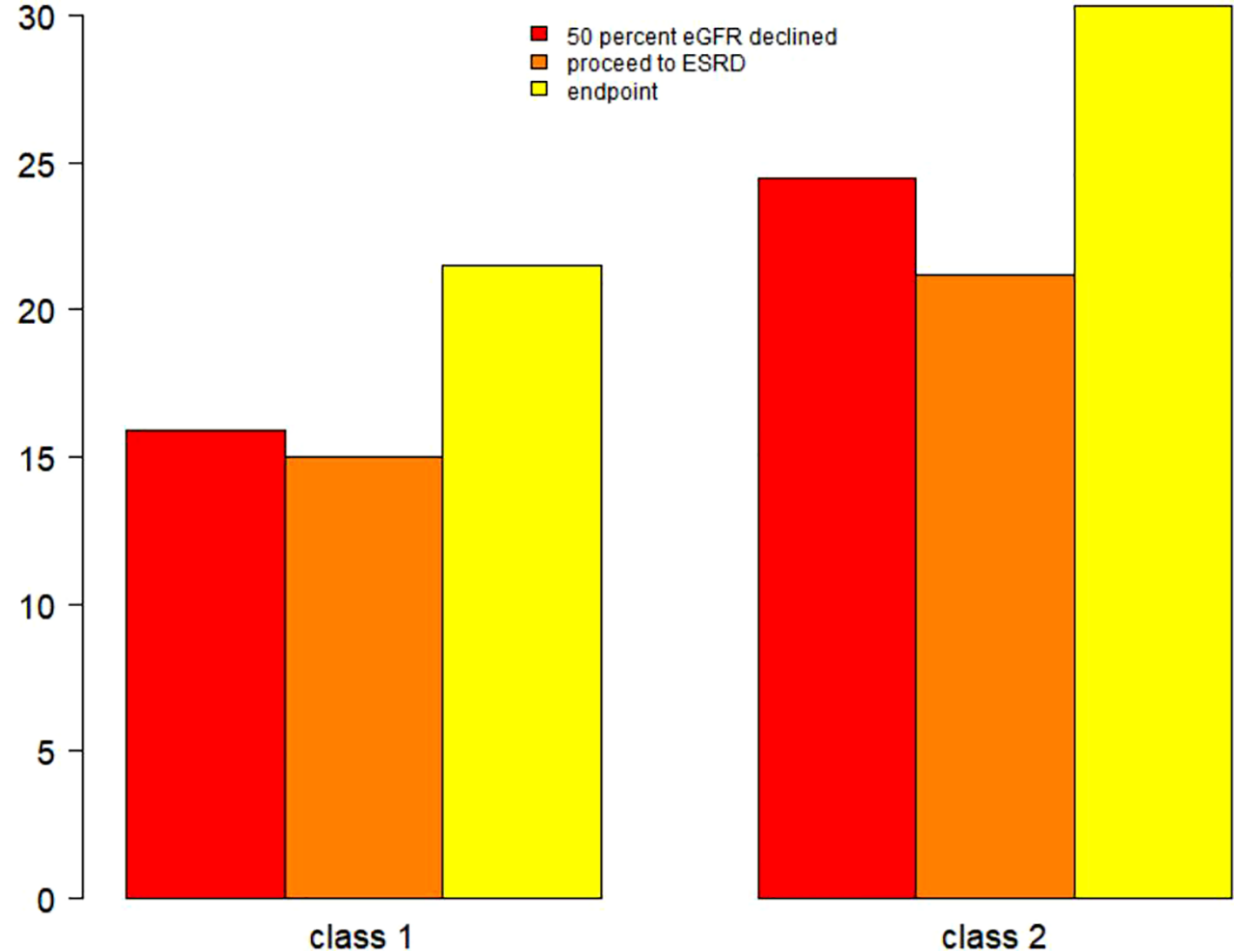
Renal endpoints in 2-class model according to latent class analysis. This figure indicates that, regardless of whether it is a 50% decline in eGFR, progression of kidney function to ESRD, or the incidence of composite renal endpoint events, patients in Category 2 have a higher incidence compared to those in Category 1.

During the follow-up period, 126 participants (21.50%) in Class 1 and 83 participants (30.29%) in Class 2 progressed to composite endpoint events. Specifically, 93 patients (15.87%) in Class 1 and 67 patients (24.45%) in Class 2 experienced a 50% decline in eGFR. Additionally, progression to end-stage renal disease (ESRD) occurred in 88 patients (15.02%) from Class 1 and 58 patients (21.17%) from Class 2. See **Figure 4**.

As revealed in **Table 4**, Class 2 demonstrated a 1.723-fold increased risk of renal composite endpoint events compared to Class 1 during the 3-year follow-up period (HR: 1.723, 95% CI: 1.275-2.329, P < 0.05). At 5-year follow-up, Class 2 showed a 1.596-fold increased risk (HR: 1.596, 95% CI: 1.204-2.115, P < 0.05), and at 10-year follow-up, the risk remained elevated at 1.591-fold (HR: 1.591, 95% CI: 1.205-2.100, P < 0.05).

**Table 4:** Cox regression analysis of 3-year, 5-year and 10-year risks for renal composite outcomes in a two-class model.

Exposure	Crude model (HR,95%CI,*P*)	Model I (HR,95%CI,*P*)	Model II (HR,95%CI,*P*)
3 years of follow-up			
Class 1	1.0	1.0	1.0
Class 2	1.723 (1.275, 2.329) 0.00040	1.678 (1.240, 2.270) 0.00079	1.718 (1.202, 2.455) 0.00299
5 years of follow-up			
Class 1	1.0	1.0	1.0
Class 2	1.596 (1.204, 2.115) 0.00114	1.518 (1.140, 2.020) 0.00423	1.662 (1.178, 2.345) 0.00385
10 years of follow-up			
Class 1	1.0	1.0	1.0
Class 2	1.591 (1.205, 2.100) 0.00104	1.507 (1.137, 1.997) 0.00429	1.826 (1.305, 2.555) 0.00045

Crude model: we did not adjust other covariates.

Model 1 adjusted for: age, gender.

Model 2 adjusted for: age, gender, alb,PCR, Hb, baseline eGFR.

HR, hazard ratios; CI, Confidence intervals; Ref: reference.

After adjusting for age, gender, albumin (Alb), estimated glomerular filtration rate (eGFR), hemoglobin (Hb) and protein-to-creatinine ratio (PCR), Class 2 continued to show significantly higher risks of renal composite endpoint events compared to Class 1 across all follow-up periods. The adjusted hazard ratios were 1.718 (95% CI: 1.202- 2.455, P < 0.05) at 3 years, 1.662 (95% CI: 1.178- 2.345, P < 0.05) at 5 years, and 1.826 (95% CI: 1.305- 2.555, P < 0.05) at 10 years.

**Figure 5:**
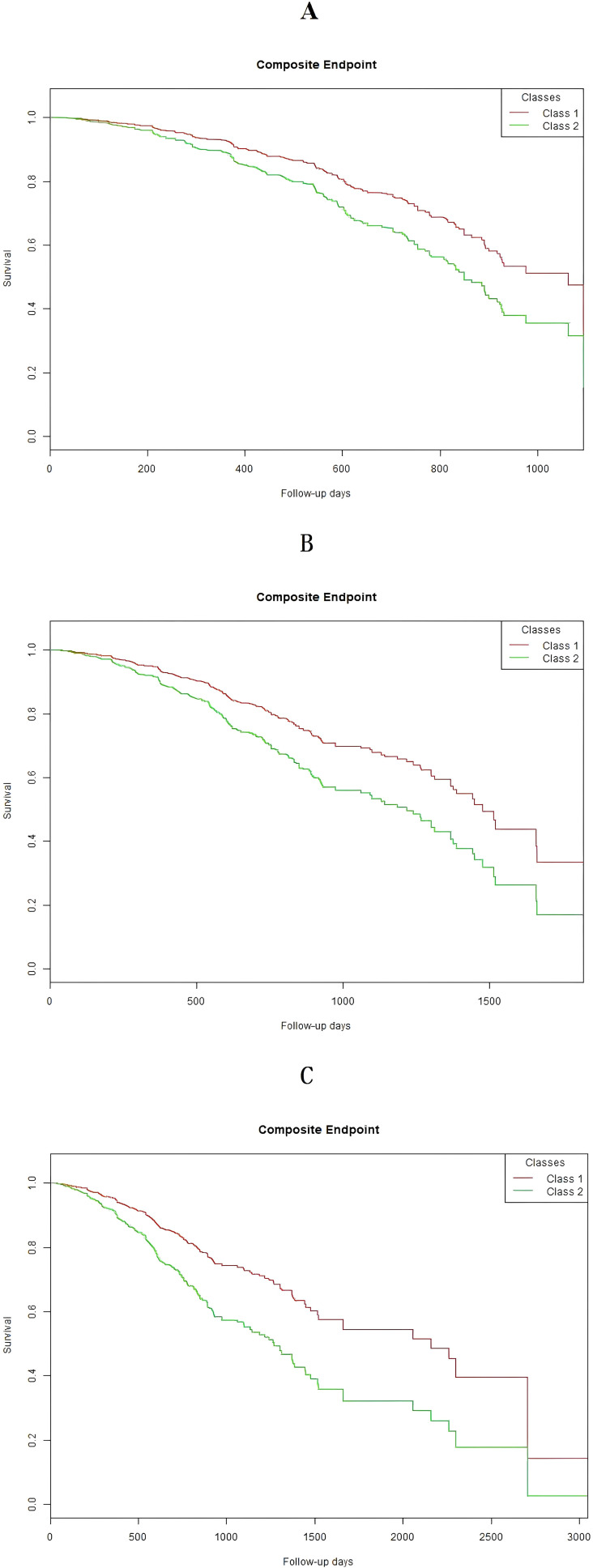
Kaplan–Meier event-free survival curve of the 2-class model. **(A)** Kaplan–Meier analysis of renal composite endpoint during 3-year follow-up (log rank, P < 0.0001). **(B)** Kaplan–Meier analysis of renal composite endpoint during 5-year follow-up (log rank, P < 0.0001). **(C)** Kaplan–Meier analysis of renal composite endpoint during 10-year follow-up (log rank, P < 0.0001).

In **Figure 5**, it presents the Kaplan-Meier curves showing the cumulative risk of renal composite endpoint events at 3-year, 5-year, and 10-year follow-up periods in the two-class model. Significant differences in the risk of renal composite endpoint events were observed between the two classes across all follow-up periods (log-rank test, p < 0.05). Class 2 demonstrated consistently higher risks of progression to renal composite endpoint events throughout the 3-year, 5-year, and 10-year follow-up periods.

## Discussion

Metabolic disorders, including glucose, lipid, blood pressure, and uric acid abnormalities, contribute to CKD progression. Given that metabolic risk factors comprise multiple indicators rather than a single factor, our study innovatively employed Latent Class Analysis (LCA) to stratify diabetic nephropathy patients from our center into distinct risk subgroups based on metabolic risk factors. These two subgroups demonstrated significant differences in baseline characteristics, with Class 2 patients exhibiting more severe metabolic disorders compared to Class 1 patients, and subsequently showing significantly different risks for renal composite endpoint events during follow-up. Class 2 patients were characterized by higher BMI, systolic blood pressure, diastolic blood pressure, glycated hemoglobin, total cholesterol, triglycerides, and low-density lipoprotein. We also found that Class 2 patients had higher levels of Hb, PCR and ACR, also a higher proportion of female patients, but lower albumin (Alb) levels compared to Class 1 patients.

Additionally, patients in Class 2 with poorer metabolic profiles are younger on average, yet have higher urinary protein excretion. Existing studies have shown that patients with early-onset type 2 diabetes typically face greater challenges in metabolic regulation. One study found that the rate of β-cell function decline in adolescents with type 2 diabetes is much faster than in adults, with an annual loss rate of 25-30% (34), compared to only 7% (35) in adults. This accelerated β-cell function decline may be one of the key drivers of metabolic dysregulation in younger patients, and the loss of β-cell function is closely associated with the occurrence and progression of diabetes complications, including diabetic nephropathy (36). Furthermore, adolescents with type 2 diabetes not only face greater challenges in terms of β-cell function but also exhibit significantly lower insulin sensitivity compared to adults, indicating more severe insulin resistance. Studies have also shown that younger-onset patients often have unfavorable fat distribution characteristics, which further exacerbates insulin resistance and leads to elevated metabolic indicators such as blood glucose, lipids, and blood pressure. Such metabolic dysregulation causes younger patients to experience more severe metabolic issues in a shorter period, thereby increasing the risk of kidney damage (37). In addition to physiological factors, patients with type 2 diabetic nephropathy typically face poorer lifestyle and management controls, especially among younger patients who fail to adopt timely and effective treatment and interventions, exacerbating their metabolic dysregulation. Therefore, despite the younger age of the patients in the second group, their more severe metabolic dysregulation might lead to a higher risk of progression to diabetic nephropathy and renal function deterioration.

During the 3-year, 5-year, and 10-year follow-up periods, Class 2 patients demonstrated higher incidence rates of composite renal endpoint events compared to Class 1 patients. Specifically, after adjusting for confounding factors, the risk of renal composite endpoint events in Class 2 was 1.718-fold (HR: 1.718, 95% CI: 1.202- 2.455, P < 0.05), 1.662-fold (HR: 1.662, 95% CI: 1.178- 2.345, P < 0.05), and 1.826-fold (HR: 1.826, 95% CI: 1.305- 2.555, P < 0.05) higher than Class 1 at 3-year, 5-year, and 10-year follow-up, respectively. Given the significantly increased risk of renal composite endpoint events in Class 2 patients across all follow-up periods, we should pay more attention to patients with severe metabolic disorders and implement more stringent lifestyle modifications or intensified medical interventions to reduce their risk of renal composite endpoint events. Our study demonstrates that Latent Class Analysis based on metabolic disorder factors is an effective method for risk stratification of renal composite endpoint events in CKD patients with type 2 diabetes.

Latent Class Analysis (LCA) is a distinctive statistical method that explains response patterns across various indicators by identifying latent (unobserved) classes (38). Its most notable characteristic is its “person-centered” research perspective, which effectively categorizes individuals with similar characteristics into different subgroups and calculates the probability of each individual belonging to a specific subgroup. Unlike traditional statistical methods (such as regression analysis) that focus on relationships between variables, LCA emphasizes the heterogeneous characteristics within populations by identifying groups with similar behavioral or characteristic patterns, thereby revealing the underlying heterogeneity within populations (39). Technically, LCA employs Full Information Maximum Likelihood (FIML) to handle missing data, which offers advantages over traditional deletion or imputation methods. In medical research, LCA has been widely applied to disease subtype classification. For instance, Calfee et al. successfully used LCA to identify two biologically distinct subtypes of Acute Respiratory Distress Syndrome (ARDS) (40), those were subtype 2 (hyperinflammatory) and subtype 1 (hypoinflammatory). Their findings revealed significant differences in responses to PEEP (Positive End-Expiratory Pressure) strategies and fluid management approaches between subtypes. This enables early identification of high-risk patients to guide individualized treatment plans, improve prognostic accuracy, support precision medicine strategies in treatment, and potentially enhance disease outcomes through optimized treatment protocols. However, the application of LCA in predicting renal outcomes among CKD patients with T2DM remains relatively limited. Our study innovatively applies LCA to identify risk stratification for renal outcomes in T2DM associated CKD. Unlike traditional studies that focus on single metabolic risk factors, we integrated a series of metabolism-related risk factors to identify high-risk subgroups, including blood pressure, blood glucose, blood lipids, body mass index, and uric acid. Importantly, we established our model without considering outcome variables, an approach that enhances result stability and reproducibility. By examining follow-up results at 3, 5, and 10 years, our study provides evidence for short-term, medium-term, and long-term risk prediction. This analysis method, based on full sample information, maximizes statistical power and reduces bias.

The latent variables in our model were associated with kidney function deterioration, consistent with previous literature reports. Our research found that metabolic factors, including systolic blood pressure, diastolic blood pressure, total cholesterol, low-density lipoprotein, triglycerides, BMI, and glycated hemoglobin, were associated with kidney function decline. Among these, hypertension is a crucial risk factor for CKD progression, with poor blood pressure control accelerating kidney function decline. Studies have shown that elevations in both systolic and diastolic blood pressure are significantly associated with the rate of kidney function decline. A cohort study of 2,769 CKD patients found that every 10mmHg increase in systolic blood pressure was associated with a 17% increased risk of kidney function deterioration (41). A cohort study of CKD patients conducted from 2003 to 2015, including 2,588 patients from a region in East Asia, demonstrated a non-linear relationship (U-shaped curve) between systolic blood pressure and CKD progression risk. Persistent abnormalities carried greater risk than intermittent ones, and blood pressure variability was also identified as a significant factor. Subgroup analysis indicated that diabetic patients had a higher risk of kidney function progression (42). Furthermore, dyslipidemia is closely associated with CKD progression. Research has demonstrated that elevated levels of total cholesterol, low-density lipoprotein, and triglycerides are significantly associated with increased risk of kidney function decline. In a prospective study involving 11,847 participants, high triglyceride levels were associated with a 26% increased risk of CKD occurrence (43). A meta-analysis including 21,411 diabetic nephropathy patients found that TC and LDL-C levels were higher in DN patients who progressed to ESRD. Controlling lipid levels, particularly TC and LDL-C, may slow the progression of DN to ESRD (44). Glycated hemoglobin (HbA1c) levels are closely associated with the progression of diabetic nephropathy. Studies have shown that each 1% increase in HbA1c is associated with a 23% higher risk of kidney function decline, and good glycemic control can significantly slow the deterioration of kidney function (45). Similarly, the progression of BMI is also closely related to the progression of kidney function (46). The relationship between metabolic factors and kidney function progression involves complex mechanisms, including RAAS system activation due to insulin resistance, promotion of oxidative stress, induction of endothelial dysfunction, and increased expression of inflammatory factors. Additionally, hemodynamic changes, adipose tissue factor-mediated injury, oxidative stress and mitochondrial dysfunction, and systemic inflammation all play roles in this process (47–49).

Evidence suggests a close association between hyperuricemia and metabolic syndrome (50–53). Therefore, in this study, we used hyperuricemia as an important marker of metabolic status in patients with diabetic nephropathy, alongside blood glucose, lipids, blood pressure, and BMI, as criteria for metabolic grouping. However, in our study, after grouping based on metabolic-related indicators through Latent Class Analysis (LCA), we found no statistically significant difference in serum uric acid levels between the two groups. Possible reasons for this include: First, serum uric acid levels are significantly influenced by renal excretory function. In a CKD cohort where baseline eGFR is similar across latent classes, uric acid levels may be uniformly elevated, reducing its ability to distinguish between different groups. In addition, uric acid is affected by various confounding factors such as diuretic use, urate-lowering medications, dietary habits, and genetic background. These factors may be unevenly distributed or inadequately measured in our cohort, potentially masking the relationship between uric acid and other metabolic factors. In our study, LCA classified participants by identifying indicators with greater between-group differences (blood pressure, lipids, glucose, and BMI), so even if uric acid is biologically relevant, its contribution to the classification may have been limited. Lastly, the single baseline measurement of uric acid did not reflect its changes over time, limiting our ability to further explore its role in the progression of renal decline. Therefore, the lack of significant differences in serum uric acid between the groups in our study does not contradict prior findings but more likely reflects cohort characteristics, confounding factors, and measurement limitations. Future sensitivity analyses, such as stratification by CKD stage, exclusion of patients on urate-lowering medications or diuretics, or using eGFR-adjusted uric acid residuals, may help further clarify the relationship between uric acid and renal function progression.

Our study has several notable strengths: 1) This is the first study to employ Latent Class Analysis (LCA) based on baseline variables to identify high-risk subpopulations among patients with T2DM associated CKD. Unlike previous literature that analyzed individual metabolic indicators’ relationships with prognosis, our approach integrated multiple metabolic indicators to classify patients into distinct subgroups. This method better reflects patients’ overall metabolic status and provides more comprehensive risk assessment, potentially revealing patterns that traditional analytical methods might miss. Traditional risk stratification methods using single or few indicators may overlook patients’ overall characteristics and fail to identify complex relationships between metabolic abnormalities and CKD progression. By identifying different patient subtypes, this approach provides a foundation for personalized treatment and helps understand specific risk factors and prognostic differences among subgroups. 2) Based on model fitting indices and clinical judgment, we selected a two-class model, which represents the most parsimonious model while maintaining accuracy. 3) We utilized line graphs to illustrate differences in continuous variables between classes and bar charts to demonstrate differences in categorical variables, enhancing the visualization of our findings. 4) Our study promotes the application of Latent Class Analysis in risk prediction, demonstrating its utility in clinical research. 5) As a retrospective cohort study, selection and observation biases were minimized through appropriate study design and methodology. 6) Our extended follow-up period allowed us to evaluate short-term, medium-term, and long-term renal outcomes, maximizing our risk prediction capabilities and providing a comprehensive assessment of disease progression.

Despite the strengths of this study, several limitations should be acknowledged: First, our findings are derived from a single-center cohort, and as such, the generalizability of the results may be limited. The metabolic characteristics and progression trajectories of chronic kidney disease (CKD) may vary across different populations and regions. Therefore, our conclusions warrant external validation through multi-center, prospective studies to assess their applicability to broader populations. Second, although we adjusted for multiple metabolic-related covariates in our analysis, the retrospective design of the study may leave room for unmeasured confounders. These unmeasured factors could still influence the observed associations, introducing potential bias into our findings. Third, the metabolic indicators in our study were only measured at baseline, which limits our ability to capture their dynamic changes over time. This static approach may not reflect the natural fluctuations of these indicators, and the absence of key variables, such as waist circumference and fasting blood glucose, may have affected the accuracy of our risk stratification. Additionally, without tracking these indicators during follow-up, we were unable to assess their evolving role in CKD progression. Future studies incorporating dynamic measures or time-dependent variables could provide a more comprehensive understanding of metabolic changes and renal outcomes.

## Conclusion

Using Latent Class Analysis, we identified two subpopulations of T2DM associated CKD with different risks of renal outcome events during 3-year, 5-year, and 10-year follow-up periods, based on metabolism-related factors including blood pressure, blood glucose, blood lipids, and body mass index. During the 3-year, 5-year, and 10-year follow-up periods, Class 2 subgroup demonstrated 1.718-fold, 1.662-fold, and 1.826-fold higher risks of composite renal endpoint events compared to Class 1 subgroup, respectively. By integrating multiple metabolic indicators to classify patients into distinct subgroups, our approach better reflects patients’ overall metabolic status and provides more comprehensive risk assessment, potentially revealing patterns that traditional analytical methods might miss. Our study, through identifying different patient subtypes, helps understand specific risk factors and prognostic differences among subgroups, thereby providing a foundation for personalized treatment approaches.

## Data Availability

The original contributions presented in the study are included in the article/**Supplementary Material**. Further inquiries can be directed to the corresponding authors.
