# Supplementary material for: Stratifying metabolic-related risk factors using latent class analysis to explore the risk of renal composite endpoints in patients with type 2 diabetes mellitus and associated chronic kidney disease
Source: Front Endocrinol (Lausanne). 2026 Jan 5;16:1599024. doi: 10.3389/fendo.2025.1599024 (PMC12813628; doi:10.3389/fendo.2025.1599024)
Supplement: Supplementary file 1 [file Table1.docx]

**Stratifying Metabolic-Related Risk Factors Using Latent Class Analysis to Explore the Risk of Renal Composite Endpoints in Patients with type 2 diabetes mellitus and associated chronic kidney disease.**

Xiaojie Chen***^1^** Weiting He^2^ Danfeng Liu^2^  Runli Jia^2^ Yaxi Zhu^2^ Haofei Hu^1^ Ricong Xu^1^ Ming Ku^1^  Hanchen Hou^2^ Xuan Zhao^2^ Qijun Wan***^1^** Wenjian Wang***^2^**

^1^Department of Nephrology, the First Affiliated Hospital of Shenzhen University, Shenzhen Second People’s Hospital, Shenzhen, Guangdong, China 518000

^2^Department of Nephrology, Guangdong Provincial People’s Hospital, Guangdong Academy of Medical Sciences, Southern Medical University, Guangzhou, Guangdong, China 510080

**Correspondence:**

Qijun Wan, Department of Nephrology, Shenzhen Second People’s Hospital, No.3002 Sungang Road, Futian District, Shenzhen 518000, Guangdong Province, China.

Email: [yiyuan2224@sina.com](mailto:??????????@126.com).

Wenjian Wang, Department of Nephrology, Guangdong Provincial People’s Hospital, Guangdong Academy of Medical Sciences, Southern Medical University, 106 Zhongshan Er Road, Main Building, Room 1436, Guangzhou, Guangdong, 510080, China.

Tel +86 (20)83827812-61421, Email wangwenjian@gdph.org.cn

Table 1A Posterior probabilities in Three-class Model.

| Class | 1 | 2 | 3 |
| --- | --- | --- | --- |
| 1 | 0.9053 (0.5108 - 0.9999) | 0.0677 (<0.0001 - 0.4939) | 0.0483 (<0.0001 - 0.4656) |
| 2 | 0.0454 (<0.0001 - 0.4720) | 0.9273 (0.4944 - 1.0000) | 0.0047 (<0.0001 - 0.4833) |
| 3 | 0.0493 (0.0001 - 0.4892) | 0.0050 (<0.0001 - 0.2698) | 0.9470 (0.5167 - 1.0000) |

Table 1B Posterior probabilities in Four-class Model.

| Class | 1 | 2 | 3 | 4 |
| --- | --- | --- | --- | --- |
| 1 | 0.9435 (0.5034 - 1.0000) | 0.0110 (<0.0001 - 0.4479) | 0.0515 (<0.0001 - 0.4913) | 0.0026 (<0.0001 - 0.2826) |
| 2 | 0.0156 (<0.0001 - 0.3128) | 0.8931 (0.3919 - 0.9988) | 0.0510 (<0.0001 - 0.4805) | 0.0584 (<0.0001 - 0.4567) |
| 3 | 0.0388 (<0.0001 - 0.4966) | 0.0440 (<0.0001 - 0.4900) | 0.8975 (0.5027 - 0.9999) | <0.0001 (<0.0001 - 0.0023) |
| 4 | 0.0021 (<0.0001 - 0.1314) | 0.0518 (<0.0001 - 0.4958) | <0.0001 (<0.0001 - 0.0012) | 0.9389 (0.5388 - 1.0000) |

Table 1C Posterior probabilities in Five-class Model.

| Class | 1 | 2 | 3 | 4 | 5 |
| --- | --- | --- | --- | --- | --- |
| 1 | 0.9138 (0.4119 - 1.0000) | 0.0298 (<0.0001 - 0.4494) | 0.0055 (<0.0001 - 0.4658) | 0.0379 (<0.0001 - 0.4520) | <0.0001 (<0.0001 - <0.0001) |
| 2 | 0.0310 (<0.0001 - 0.3956) | 0.8600 (0.4141 - 1.0000) | 0.0114 (<0.0001 - 0.4741) | 0.0739 (<0.0001 - 0.4594) | 0.0244 (<0.0001 - 0.4101) |
| 3 | 0.0009 (<0.0001 - 0.1526) | 0.0120 (<0.0001 - 0.3748) | 0.9340 (0.5010 - 1.0000) | 0.0026 (<0.0001 - 0.1283) | 0.0334 (<0.0001 - 0.4272) |
| 4 | 0.0543 (<0.0001 - 0.4295) | 0.0656 (<0.0001 - 0.4778) | 0.0005 (<0.0001 - 0.0204) | 0.8500 (0.3958 - 0.9991) | 0.0317 (<0.0001 - 0.4205) |
| 5 | <0.0001(<0.0001 - <0.0001) | 0.0326 (<0.0001 - 0.4075) | 0.0486 (<0.0001 - 0.4990) | 0.0390 (<0.0001 - 0.4958) | 0.9105 (0.5589 - 0.9998) |
